# Vitrification and rapid rewarming of precision‐cut liver slices for pharmacological and biomedical research

**DOI:** 10.1002/btm2.70045

**Published:** 2025-07-17

**Authors:** Srivasupradha Ramesh, Joseph Sushil Rao, Bat‐Erdene Namsrai, Benjamin Fisher, Diane K. Tobolt, Michael Megaly, Michael L. Etheridge, Timothy L. Pruett, Davis Seelig, Paari Murugan, Bashar Aldaraiseh, Erik B. Finger, John C. Bischof

**Affiliations:** ^1^ Department of Mechanical Engineering University of Minnesota Minneapolis Minnesota USA; ^2^ Department of Surgery University of Minnesota Minneapolis Minnesota USA; ^3^ Schulze Diabetes Institute University of Minnesota Minneapolis Minnesota USA; ^4^ Department of Veterinary Clinical Sciences University of Minnesota St Paul Minnesota USA; ^5^ Department of Laboratory Medicine and Pathology University of Minnesota Minneapolis Minnesota USA; ^6^ Institute for Engineering in Medicine University of Minnesota Minneapolis Minnesota USA

**Keywords:** cryopreservation, liver slice, vitrification

## Abstract

High‐throughput in vitro pharmacological toxicity testing is essential for drug discovery. Precision‐cut liver slices (PCLS) provide a robust system for screening that is representative of the complex 3D structure of the whole liver. However, PCLS are not available as off‐the‐shelf products. Cryopreservation could solve this bottleneck by effectively preserving PCLS indefinitely until their time of use. With cryopreservation, slices could be shipped to laboratories without access to fresh tissue or used in planned experiments independent of surgical schedules. Here, we explore an “ice‐free” cryopreservation approach called vitrification and focus on culturing and assessing PCLS for 3 days post‐vitrification and rewarming, given that most acute drug toxicity tests are conducted over 24 h. Rat liver slices were diffusively loaded with a cryoprotective agent (CPA) cocktail consisting of ethylene glycol and sucrose. The CPA‐loaded PCLS were placed on a polymer cryomesh, vitrified in liquid nitrogen, and rapidly rewarmed in CPA. The vitrified and rewarmed PCLS were subsequently cultured in serum‐free media for 3 days. The cryopreserved PCLS maintained high viability, morphology, function, enzymatic activity, and drug toxicity response. Results show that the vitrified PCLS perform comparably to untreated controls and significantly outperform conventionally cryopreserved PCLS in all assessments (*p* < 0.05).

**Abbreviations**
ANOVA: analysis of variance
AO/PI: acridine orange/propidium iodide
APAP: *N*‐acetyl‐para‐aminophenol (acetaminophen)
ATP: adenosine triphosphate
CCR: critical cooling rate
CPA: cryoprotective agent
CWR: critical warming rate
DAB: 3,3′‐diaminobenzidine
DMSO: dimethyl sulfoxide
EG: ethylene glycol
ETFE: ethylene tetrafluoride
HSD: honestly significant difference
IACUC: Institutional Animal Care and Use Committee
IQR: interquartile range
PCLS: precision‐cut liver slices
R&D: research and development
TUNEL: terminal deoxynucleotidyl transferase dUTP nick end labeling
UW: University of Wisconsin
VR: vitrification and rewarming

**Translational Impact Statement**

Cryopreservation of precision‐cut liver slices (PCLS) through vitrification would allow for the development of an “off‐the‐shelf” cryo supply chain of human PCLS that are stored in a repository and available for on‐demand shipping for research. The translation of this technology would allow PCLS to become a scalable, reproducible, wide‐ranging, and population‐representative source of tissue that can accurately mimic in vivo conditions of the human liver, thereby revolutionizing our methods of drug discovery and development.

## 1 INTRODUCTION

The costs of bringing a new pharmaceutical to market range between $2.6B and $6.7B, when including capital and failure costs.^1^ The decrease in successful R&D outcomes within the biopharma industry poses challenges to the global healthcare ecosystem by increasing price pressure and extending timeframes to deliver life‐saving treatments to patients.

Current in vitro drug testing and discovery models are mostly animal‐derived and cell‐based, and they fail to accurately predict the toxicity and efficacy of drugs in humans, leading to failures during clinical trials.^2^ In vitro drug metabolic studies use less complex testbeds, which do not adequately capture the complex dynamics of the multicellular mechanisms of the human body.^3^, ^4^ This pushes us toward an R&D crossroads where the need for accurate and efficient testbeds for biomedical research is critical.

Hepatotoxicity is the most common cause of pharmaceutical failure and withdrawal from the market. Ideally, drug metabolism is studied in vivo, but this becomes prohibitive in terms of cost, accessibility of samples, and ethical concerns.^5^ Alternatively, precision‐cut liver slices (PCLS) can act as a well‐characterized model. PCLS maintain in vivo architecture, cell populations, and function of the whole organ. Since their introduction 30 years ago, PCLS studies have demonstrated the ability to mimic drug pathways in humans for drug efficacy and toxicity studies.^6^, ^7^ PCLS can also be created from diseased livers, making them excellent in vitro disease models for a wide range of biomedical research. Arguably, the biggest barrier to PCLS use is the lack of standardized protocols for preserving PCLS like those that exist for cells.^6^ Improving protocols for preserving, storing, and shipping PCLS would broaden access for a variety of applications. However, like organs, the viability of PCLS rapidly degrades within hours in cold storage. Under culture, PCLS can last for only a few days, but viability and function decline with time in culture.^8^

Cryopreservation, or the storage of biosystems at ultralow temperatures, could enable the on‐demand supply of PCLS for pharmacotoxicology and other biomedical applications. However, conventional freezing cryopreservation protocols using slow cooling in dilute concentrations of dimethyl sulfoxide (DMSO) have proven unreliable for PCLS. While slow freezing with ice is commonly employed in cell suspensions,^9^ it fails in complex tissues where ice formation disrupts cellular and tissue structures, severely compromising viability and function.^10^ Indeed, the best PCLS freezing protocols published have resulted in only 70% viability after 4 h in culture.^10^

**FIGURE 1:**
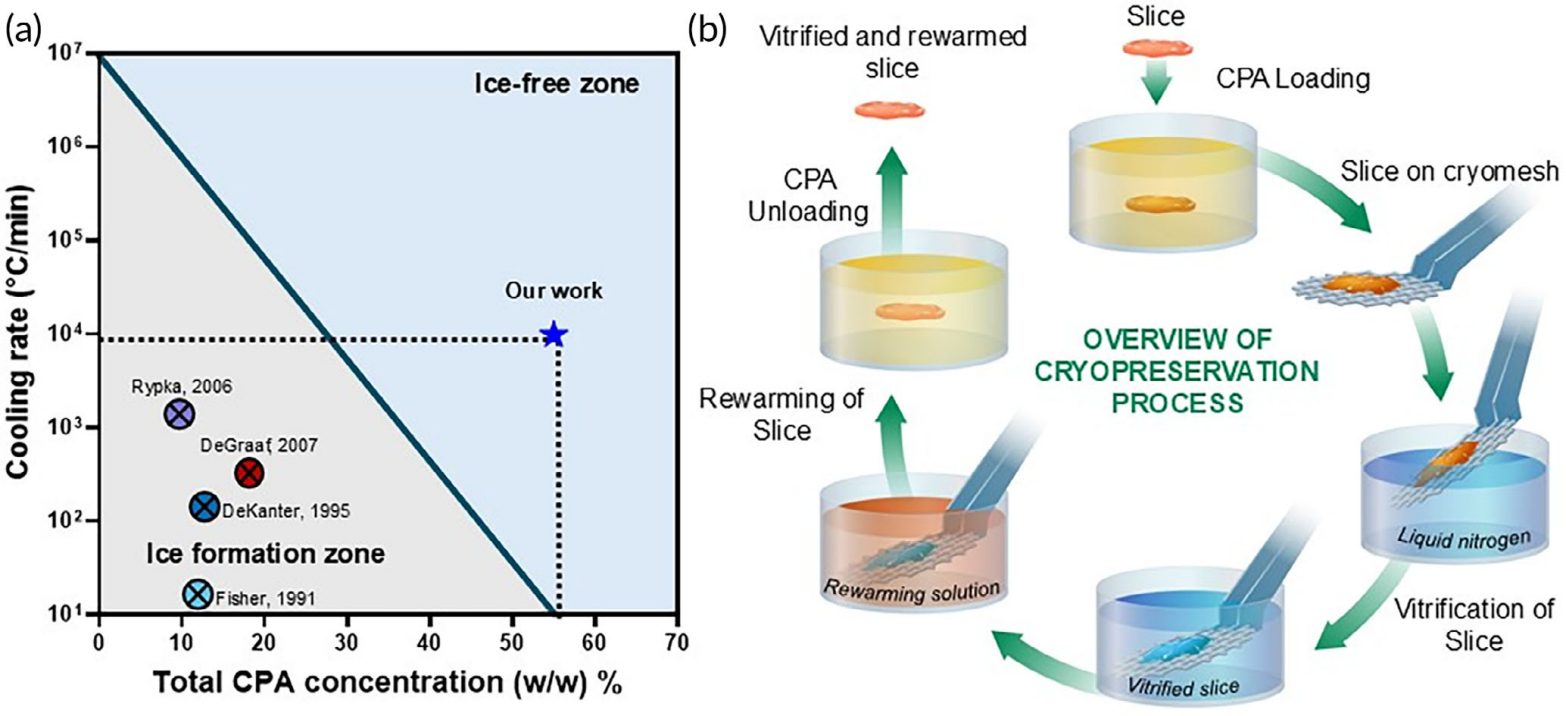
Overview of precision‐cut liver slices (PCLS) cryopreservation. (a) Previous works in the cryopreservation of PCLS report cooling rates that would lead to damaging ice formation.^10^, ^12^, ^13^, ^14^ Our work uses high concentration cryoprotective agents and rapid cooling and rewarming to achieve ice‐free vitrification. (b) Schematic diagram of the steps in the process for vitrification and rewarming using the cryomesh. CPA cryoprotective agent.

An alternative to slow freezing is vitrification, which uses high concentrations of cryoprotective agents (CPAs) and high cooling rates to form a glass‐like “ice‐free” state (Figure 1a). We posit that vitrification would allow for the storage of PCLS at cryogenic temperatures with minimal to no loss in their viability, function, and enzymatic activity. To successfully vitrify, we must cool the PCLS faster than the CCR (critical cooling rate required to avoid ice formation) and rewarm them faster than the CWR (critical rewarming rate required to avoid ice formation).^11^ Cryopreservation of PCLS by vitrification requires a delicate balance of having sufficient concentration of CPA to avoid ice formation under the achieved cooling and rewarming rates, while minimizing toxicity from the CPA.

In this study, we used rat liver slices as a representative biological model. We chose a CPA based on previous literature^15^, ^16^ and performed systematic studies to optimize the CPA loading and unloading conditions. To support rapid cooling and rewarming, we utilized a cryomesh system that we previously used to successfully cryopreserve islets^17^ and other organisms.^18^ Finally, we assessed PCLS viability, metabolism, function, enzymatic activity, and drug response over 3 days in culture. The overview of our methodology is presented in Figure 1b.

## 2 METHODS

### 2.1 Ethics statement

JCB, EBF, and MLE disclose equity in a start‐up that is commercializing the cryomesh platform technology and related intellectual property (NorthStar Cryo, Inc.).

### 2.2 Preparation of PCLS

Livers were recovered from 2.5‐3‐month‐old Sprague–Dawley rats (Charles River Labs, Wilmington, MA) and sliced into 5 mm wide and 250 μm thick slices as described in the Supporting Information S1. The University of Minnesota IACUC approved the study. All data are from three biologically replicate experiments (*N* = 3) with four slices (*n* = 4) per biological replicate for each experimental condition unless stated otherwise.

### 2.3 Culturing PCLS using mesh in six‐well plate

The slices were cultured in six‐well tissue culture plates with 1 mL of media per slice per well on nylon meshes with 0.3 mm filament thickness and 500 μm pore size under normoxic conditions in a 37°C incubator. This maintained the media height above the tissue at 0.45 mm. The media composition is presented in Supporting Information S1.

### 2.4 Vitrification and rewarming of PCLS

**FIGURE 2:**
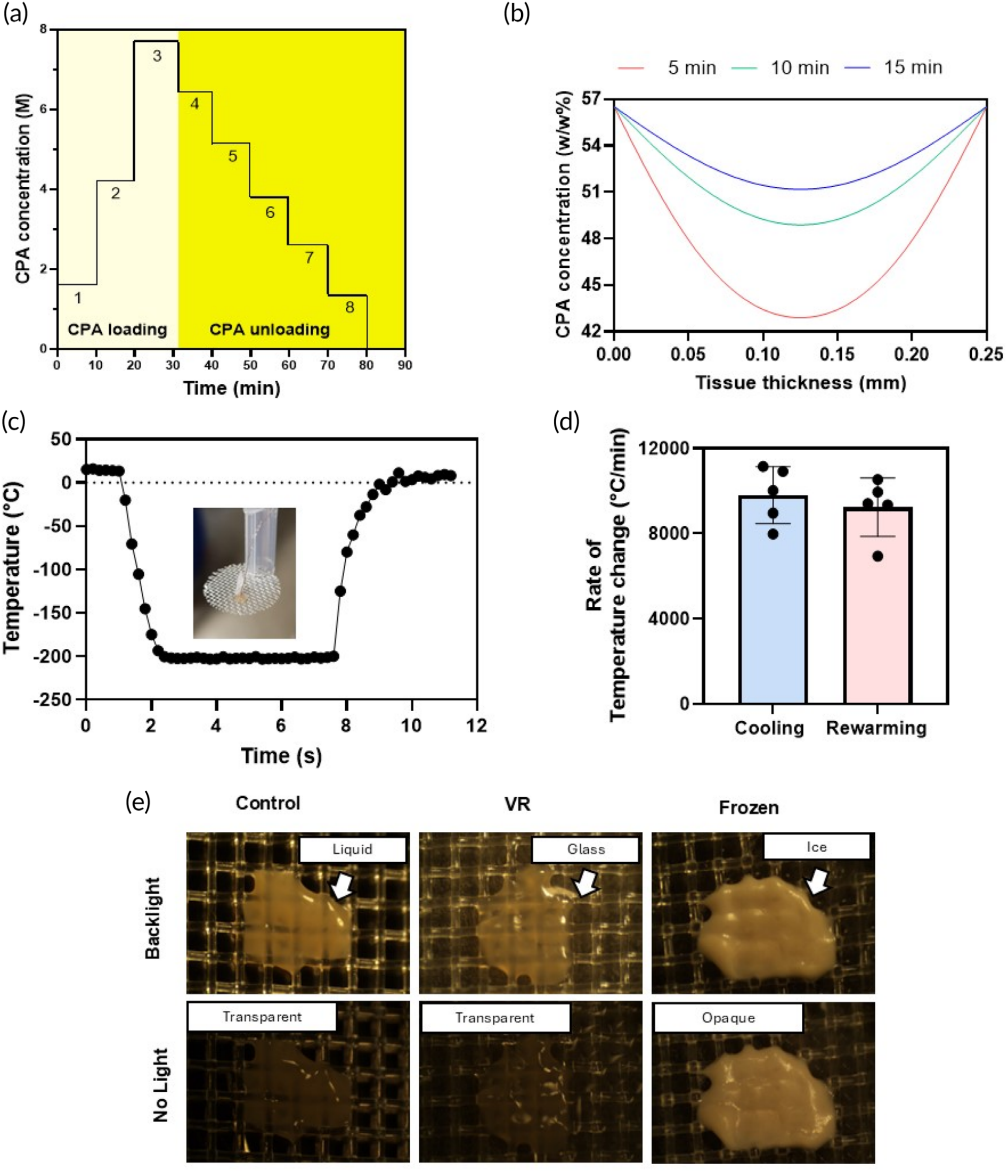
Vitrification characteristics of precision‐cut liver slices (PCLS). (a) The cryoprotective agent (CPA) loading and unloading profile was an initial three steps of EG followed by a final step of 40% (v/v) ethylene glycol and 0.6 M sucrose. Post‐vitrification, we unloaded the CPA in six steps of decreasing sucrose concentration and a final carrier solution‐only step. (b) The COMSOL model estimated the total CPA concentration throughout the tissue following loading steps of either 5, 10, or 15 min. (c) Representative thermometry of the PCLS taken during vitrification and rewarming (VR) on the cryomesh. Inset is a picture of the thermometry setup. (d) Cooling and rewarming rates measured from thermometry. (e) Visualization of ice in the PCLS. Control and VR are transparent, indicating no ice, and the frozen slice is opaque due to ice formation.

PCLS were transferred to ethylene tetrafluoride (ETFE) cryomeshes with a monofilament diameter of 25 μm and 500 μm pore size. The PCLS were loaded with the CPA (diluted in modified University of Wisconsin [UW] carrier solution) in steps as follows: 10% ethylene glycol (EG) for the first step, 25% EG for the second step, followed by the final CPA concentration of 40% EG + 0.6 M sucrose, as indicated in Figure 2a. Once loaded with CPA, the PCLS were placed on the cryomesh and convectively vitrified by vertical immersion in liquid nitrogen to reduce Leidenfrost boiling, as previously described.^18^ The PCLS were then convectively rewarmed by immersion in rewarming solution (1 M sucrose) at room temperature, followed by unloading the CPA from the slices at 4°C in steps, starting with (0 EG) and 1 M sucrose, followed by 0.7, 0.525, 0.35, and 0.175 M sucrose, with the final step being the carrier solution, as indicated in Figure 2a.

### 2.5 COMSOL model

COMSOL Multiphysics 5.6 (COMSOL, Burlington, MA) was used to model the CPA diffusion in PCLS. The preset properties for human liver were used for the model with heat capacity Cp = 3540 J/(kg K), density = 1079 kg/m^3^, thermal conductivity = 0.52 W/(m K).^19^, ^20^, ^21^, ^22^, ^23^, ^24^ A 0.25 mm square geometry was used to represent the tissue, as the characteristic length of interest is the thickness of the tissue. The Transport of Concentrated Species physics model was used to estimate the diffusion of CPA components for loading times. A mixture average diffusion model was used, and the Maxwell‐Stefan diffusivities for the CPA components were calculated using the formula reported by Yu et al.^25^ with the free diffusion coefficients of the components calculated using the Stokes‐Einstein formula. The species diffusivity was calculated as follows: EG‐Sucrose ➔ 5.226e−12 m^2^/s, EG‐carrier ➔ 3.85e−11 m^2^/s and sucrose‐carrier ➔ 5.618e−12 m^2^/s. The coefficient for sucrose was calculated using the viscosity values provided by the National Bureau of Standards (Circular 440)^26^ at 75 (%w/w) at 5°C. The temperature in the model was set to 277 K with a mixture density of 1140 kg/m^3^ and molar mass of EG, sucrose, and carrier to 0.062, 0.34, and 0.018 kg/mol, respectively. An initial value of 4.2 M EG was set with 0 M sucrose, and an inflow of 7.1 M EG and 0.6 M sucrose was set as the boundary conditions on both ends of the tissue to estimate the final step time of CPA diffusion required for successful vitrification. The concentrations were then obtained for different loading step times of 5, 10, or 15 min. This is represented in Figure 2b.

### 2.6 Functional assays and cytochrome P450 1A1 live tissue imaging and quantification

Assays for urea, albumin, and ATP were performed according to manufacturers' instructions, the details of which are provided in Supporting Information S1. For live imaging of cytochrome P450 1A1 (CYP1A1) activity, CYP1A1 was induced in the slices using 25 μM β‐naphthaflavone for 24 h. PCLS were then incubated with 20 μM 7‐ethoxyresorufin and 25 μM dicumarol for 10 min, followed by imaging or quantification. CYP1A1 cleaves 7‐ethoxyresorufin to fluorescent resorufin that can be imaged and quantified.^27^ The slices were imaged using an excitation wavelength of 561 nm laser in a Nikon A1RMP+ microscope. For quantification, the slices were placed in a microplate reader and imaged for 30 min in kinetic mode using 535/595 nm filters at 37°C. Additional details are provided in Supporting Information S1.

### 2.7 Acetaminophen drug study with cryopreserved slices

*N*‐Acetyl‐para‐aminophenol (acetaminophen) (APAP) was prepared in culture media with supplements (details in Supporting Information S1) in increasing concentrations: 0, 1, 5, 10, 20, and 50 mM. Slices were then exposed to the APAP culture media for 24 h, after which the media was replaced with media containing no APAP and cultured for 2 more days.

### 2.8 Statistics

Statistical analysis was performed in R version 4.3.2 (R Foundation for Statistical Computing, Vienna, Austria). Normality was established using the Shapiro–Wilk test to compare continuous variables, and homogeneity of variance was assessed using Levene's test. For normally distributed group comparisons, ANOVA testing with pairwise post hoc *t*‐test for single comparisons or Tukey HSD test for multiple comparisons was used. Non‐normal variables were tested using the non‐parametric Kruskal–Wallis and pairwise Wilcox (Mann–Whitney *U*) or Dunn's tests for individual group comparison. Grubb's test and IQR test were used to screen and censor outlier data. The Benjamini–Hochberg method was used to adjust for multiple comparisons. A *p*‐value less than 0.05 was taken to be statistically significant (*) (*p* < 0.005 represented by **, *p* < 0.0005 by ***, *p* < 0.00005 by ****). Continuous data are presented as mean ± standard deviation. Only statistically significant differences are shown in the figures.

### 2.9 Additional materials and methods

Full details of the materials, assaying methods, culture conditions, and other experimental procedures are available in Supporting Information S1.

## 3 RESULTS

### 3.1 Vitrification and rewarming of PCLS

This study tested cryopreservation and post‐rewarming viability, function, enzymatic activity, and drug response in 250 μm thick × 5 mm diameter PCLS from Sprague–Dawley rats as a model system. We vitrified the liver slices using a cryoprotectant cocktail consisting of 40% (v/v) of EG and 0.6 M sucrose, initially developed for the cryopreservation of hepatocytes^16^, which we have used in whole rat livers.^15^ A modified version of UW solution was used as carrier (starch‐free with a 1 g/L concentration of PEG35k for oncotic support, as previously reported^28^).

Osmotic injury and toxicity are two of the main challenges to resolve when designing CPA loading and unloading protocols. For this study, CPA loading and unloading were performed stepwise to limit osmotic injury (Figure 2a). CPA exposure was conducted at 4°C to reduce toxicity, as we previously used in rat livers.^15^

We determined the CPA loading protocol by developing a multicomponent diffusion model in COMSOL that estimated EG and sucrose tissue concentration over time, as previously described for other permeant CPAs^25^. The model tested three step durations (5, 10, or 15 min). At 5 min, the model predicted a minimum CPA concentration of 42.8 (% w/w) in the interior of the PCLS, whereas this increased to 48.8 (% w/w) at 10 min. Our previous studies^11^ estimated that a minimum of 48 (% w/w) of CPA would be needed to successfully vitrify and rewarm without ice formation at the cooling and rewarming rates achievable by our cryomesh. Thus, a 10‐min step duration was chosen for this specific CPA loading and unloading process.

The vitrification and rewarming (VR) procedure was then experimentally validated by thermometry and visual inspection (*n* = 5). Vitrification of the slices with 10‐min loading steps indicated no visible ice (Figure 2e).

The VR of the PCLS was done on ETFE cryomeshes. Temperature measurements taken during cooling and rewarming of the slices indicated average cooling rates of 9800°C/min and rewarming rates of 9200°C/min (Figure 2c,d), which exceeded the expected CCR (24.6°C/min) and CWR (7703°C/min) at the minimum required concentration (48 w/w%) calculated from previous studies.^11^

### 3.2 Viability and morphology of cryopreserved PCLS

**FIGURE 3:**
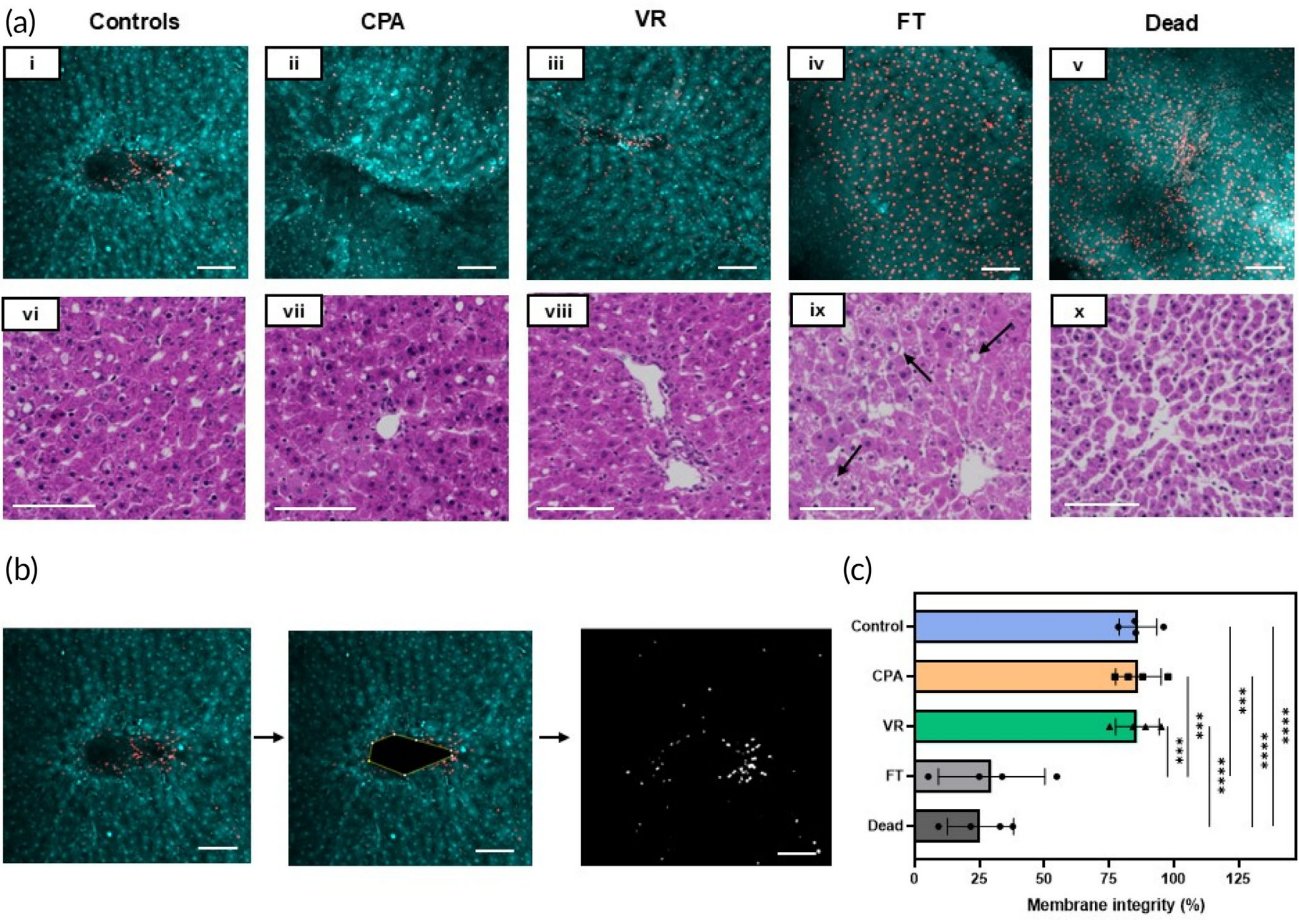
Viability and tissue morphology directly post‐cryopreservation. (a) (i–v) Acridine orange/propidium iodide (AO/PI) staining where AO‐stained cells appear green, and the PI‐stained cells are red, indicating cell membrane compromise. (vi–x) Hematoxylin and eosin staining of the slice groups just post‐rewarming. (b) The viability through membrane integrity was assessed by analyzing the number of dead cells in the given *z*‐plane by assessing areas of individual cells and comparing the number of PI‐quenched cells to the total number of cells. (c) Viability measure from AO/PI for each group. Levels of significance: ***p = 0.0001; ****p < 0.0001 (one‐way ANOVA). AO, acridine orange; CPA, cryoprotective agent; FT, slow freezing and rapid thawing; VR, vitrification and rewarming.

The viability of the slices was assessed using acridine orange/propidium iodide (AO/PI) viability stain (live cells are green in color, and dead cells are red). Fresh control groups were compared to PCLS treated with CPA‐only (CPA loaded and unloaded), VR, conventional cryopreservation (slow freezing and rapid thawing [FT] in cryovial after loading with 18% DMSO [v/v] for 30 min on ice, FT), and a negative control group (freeze‐thawed three times with no CPA, dead) (Figure 3a). Live and dead cell fractions were quantified by image analysis (Figure 3b,c).

The morphology of the slices was assessed by histologic staining (hematoxylin and eosin [H&E]), which showed that PCLS from the control, CPA, and VR groups had well‐preserved hepatocytes, microarchitecture, and portal tracts. In comparison, the FT group exhibited significant vacuolization (marked with arrows in Figure 3a, ix), and the dead group showed significantly damaged microarchitecture. These were predicted to substantially affect viability, consistent with the measures observed.

### 3.3 Culture conditions for liver slices

**FIGURE 4:**
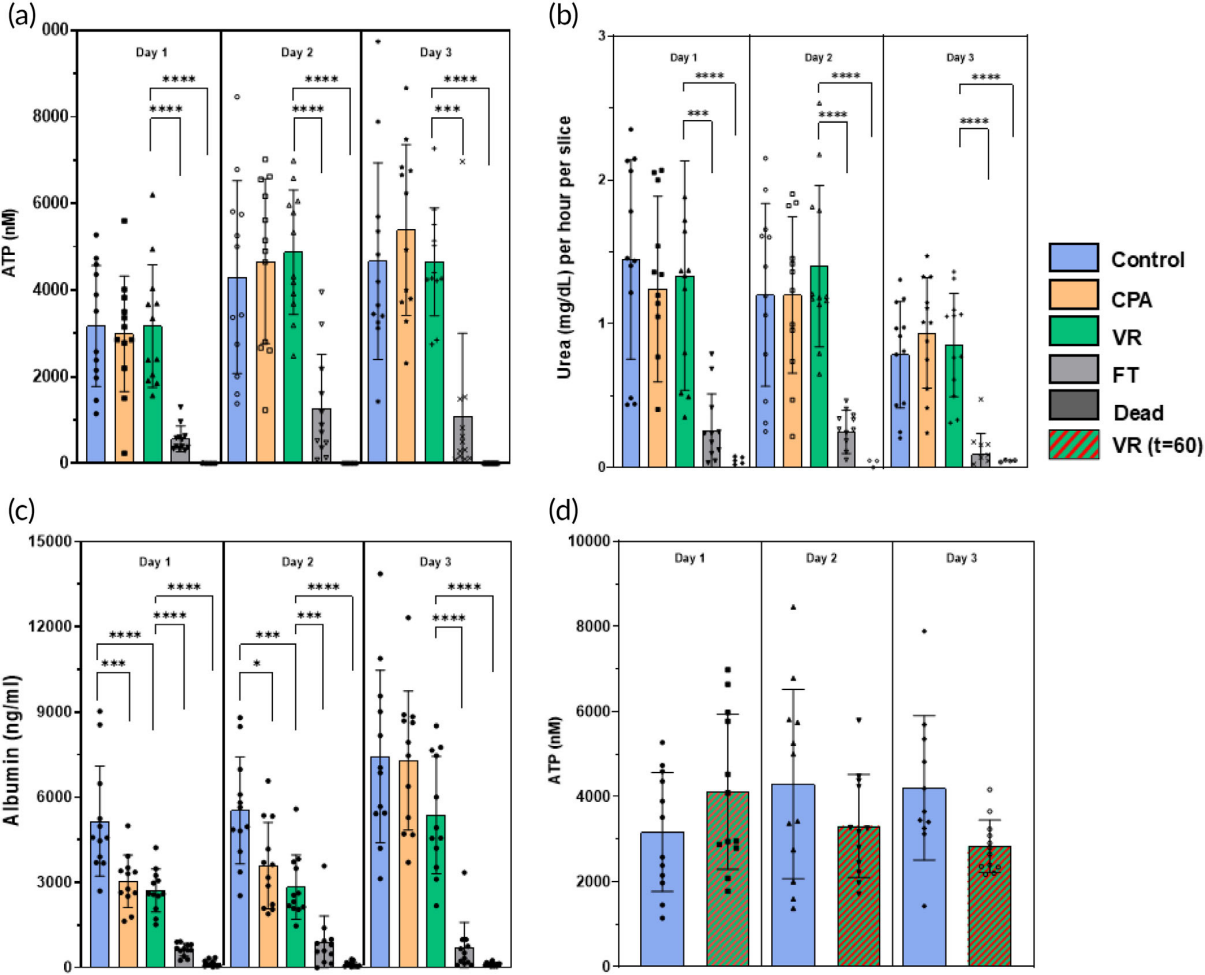
Metabolic and functional assessments of liver slices over 3 days in culture. Metabolic and functional assessments of liver slices over 3 days in culture. (a) ATP assessment. (b) Urea production. (c) Albumin synthesis. (d) ATP assessment of vitrification and rewarming (VR) group that was stored at −150°C for 60 days (*t* = 60). Levels of significance: *p < 0.05, ***p < 0.0005, and ****p < 0.00005 (Mann–Whitney *U* test for [a–c] and Kruskal–Wallis test for [d]). CPA, cryoprotective agent; FT, slow freezing and rapid thawing.

Previous attempts have been made to culture liver slices for extended periods, but for most drug discovery and testing applications, immediate acute toxicity tests are conducted within 24 h.^29^ When conducting trials for daily dose drugs, there are perspectives on how an optimal drug design for oral drugs should aim to have a half‐life of 12–48 h^30^. So, we set culturing timeframes to be 3 days for the initial development of the study as a conservative estimate for the practical window needed for drug studies. Previous studies have shown that sufficient passive oxygenation for hepatocytes can be provided by adjusting the media height above the cells, eliminating the need for high levels of external oxygenation.^31^ Therefore, we systematically studied the height of the media above the PCLS, striving to avoid supplemental external oxygenation of the media while maintaining maximum viability and function. We found that 1 mL of media was required to sustain a single slice over 24 h (200:1 media to tissue volume) and optimized the media height above the tissue to 0.45 mm. Additionally, culturing the slice on meshes enhanced oxygen diffusion from both sides. The control slices cultured directly on the meshes also did not show any difference in viability, function, or morphology compared to the VR group (Figures 3 and 4) that was cryopreserved on ETFE meshes and then transferred to nylon meshes to culture. This resulted in our ability to culture PCLS with high viability, function, and drug response for up to 3 days in culture.

### 3.4 Metabolic health and functional assessments in cryopreserved slices

We assessed the metabolic health of the PCLS by their ATP levels (Figure 4a). The ATP levels of the controls, CPA, and VR groups remained high and consistent throughout the 3 days in culture (Figure 4a). The FT group showed significantly lower ATP levels throughout the 3‐day culture period, while the dead group remained below the detection limit of the assay used (<25 nM).

Good function of the cryopreserved slices is required for effective biomedical and drug testing applications. To measure liver slice function, we analyzed urea production (Figure 4b) and albumin synthesis (Figure 4c). With urea production, the CPA‐only and VR groups behaved similarly to controls, where urea production remained consistent throughout the 3 days in culture with mean values equal to or above 0.7 mg/dL for all three groups. A slight drop in urea production was observed on Day 3 but was not statistically significant. The FT group showed significantly less urea production over all 3 days, consistent with other measurements. The dead group showed no measurable urea production.

Albumin synthesis stayed high and consistent with the control groups over all 3 days (Figure 4c). The CPA and VR groups experienced a statistically significant initial decrease in albumin synthesis compared to controls but then caught up to controls on the third day of culture. This could be due to recovery of the slices from an initial stress response on exposure to the CPA and during vitrification/rewarming, but it did not seem to hinder the ability of the slices to synthesize new albumin.^32^ An increase in albumin synthesis was seen with the controls, CPA, and VR groups when comparing Day 1 to Day 3, indicating preserved function of the CPA and VR slices. The FT and dead groups had significantly less albumin synthesis over all 3 days in culture. However, the dead controls still showed low albumin levels, possibly indicating a slow leakage of pre‐formed albumin rather than actual synthesis.

To demonstrate that the slices could be stored for extended periods of time once vitrified, we stored vitrified slices at −150°C for 60 days, rewarmed the slices, unloaded the CPA, and assessed their ATP levels (Figure 4d). The ATP levels of the stored slices were consistent across the 3 days in culture, and in comparison to controls, the stored and rewarmed slices (*t* = 60) did not show any differences in ATP on all 3 days of culture.

### 3.5 Acute testing for zonated cytochrome P‐450 activity, viability, and apoptosis

Liver slices afford the opportunity to assess the zonal activity of cytochrome P‐450 (CYP) enzymes that form the crux of xenobiotic metabolism. These enzymes are zonated, with the most activity present in Zone 3 near the central hepatic vein draining the hepatic lobule. To understand if the zonal CYP activity is observed in the VR slices, we induced CYP1A1 in the control and VR PCLS for 24 h, followed by imaging. This allowed for assessment of zonal CYP activity after cryopreservation where the tissue may undergo significant change and injury. We also quantified the activity throughout the 3 days in culture. We chose to compare the CYP activity only between the controls and the VR groups, as we posit that since even basic function is severely impaired in the FT and dead groups, enzymatic activity, apoptosis, and drug response would also be of significantly lower performance.

**FIGURE 5:**
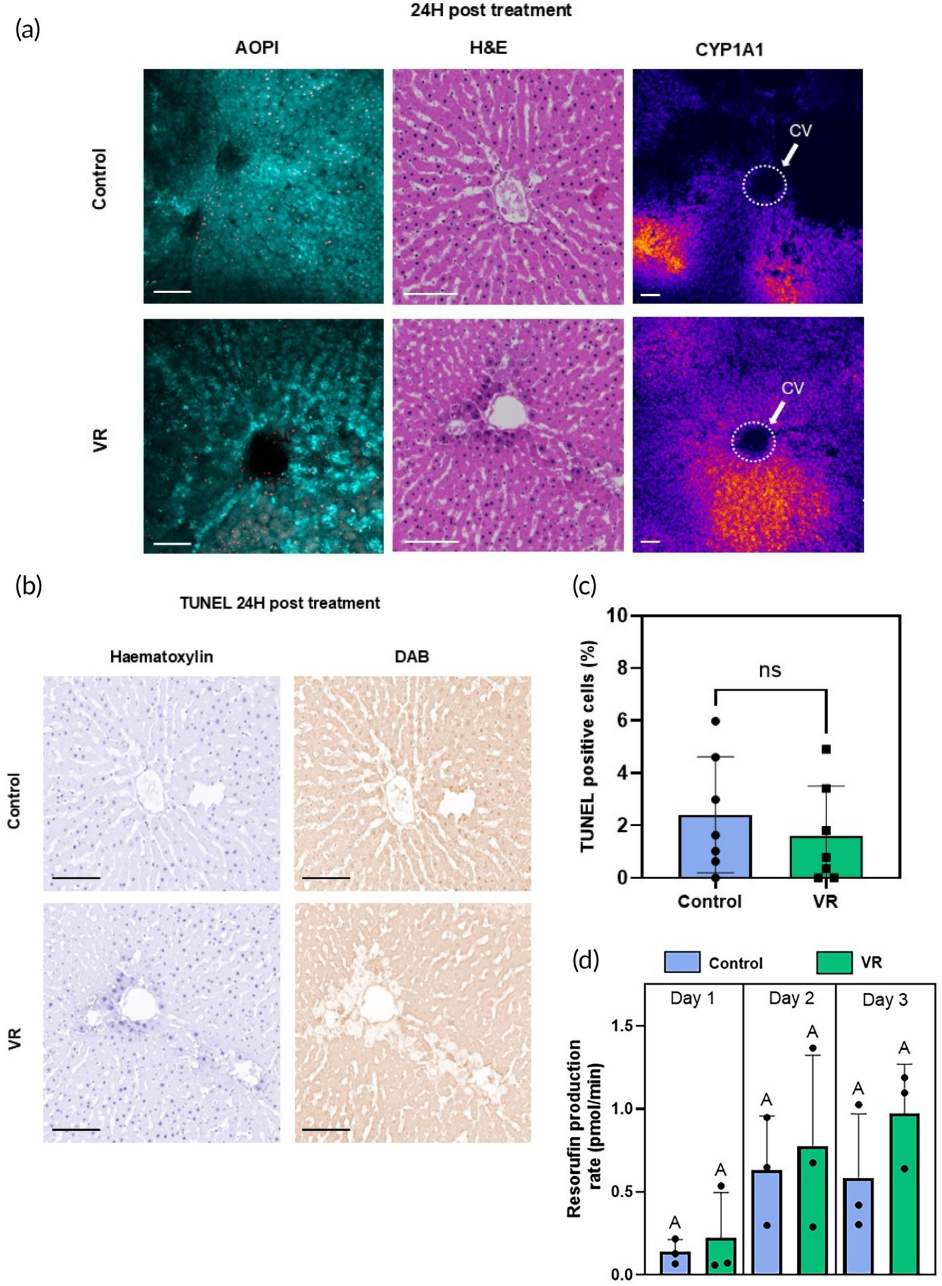
Acute injury response of slices and quantified enzymatic activity. (a) Acute evaluation of precision‐cut liver slices with acridine orange/propidium iodide (AO/PI), hematoxylin and eosin (H&E) staining, and cytochrome P450 1A1 (CYP1A1) zonated activity. (b) TUNEL staining after 24 h culture to evaluate apoptosis (or necrosis) in both groups. Hematoxylin staining (blue) shows all nuclei and 3,3′‐diaminobenzidine (DAB) stain (brown) shows TUNEL‐positive nuclei. (c) Quantification of TUNEL‐positive cells (*n* = 7). (d) Rates of resorufin production (*N* = 3, *n* = 4 where *N* indicates biological replicates and *n* indicates number of slices from a single biological replicate). The level of significance is presented by compact letter display (Kruskal–Walli's test). CV, central vein; VR, vitrification and rewarming.

By confocal imaging, the cleaved resorufin was visible as a bright signal near the central vein in the acini of the liver slices in both the control and VR groups, indicating zonated CYP activity in Zone 3. Quantification of fluorescence demonstrated that the resorufin production rate was induced and similar between control and VR PCLS over the 3‐day culture. Both assessments showed that CYP activity post‐cryopreservation was zonated in the acute 24‐h period and that activity was maintained during all 3 days in culture (Figure 5a,d).

TUNEL staining was also performed after 24 h to assess induction of apoptosis (or necrosis) (*n* = 7). The TUNEL‐positive cell fraction did not differ between VR and control PCLS (Figure 5b,c). Control and VR groups showed an average of 2.8% and 1.8% TUNEL‐positive cells, respectively, which were not statistically different.

### 3.6 Acetaminophen hepatotoxicity in fresh and cryopreserved PCLS

The most critical issue related to in vitro drug studies is the potential for hepatotoxicity, representing a major failure mechanism for pharmaceuticals. To demonstrate the use of cryopreserved PCLS as an in vitro model for drug testing, we exposed the control, VR, and dead slices to different concentrations of APAP. We chose APAP as a model drug for testing since it is well‐studied, and APAP overdose is a leading cause of hepatotoxicity, which in some cases could lead to fatal acute liver failure.^33^

**FIGURE 6:**
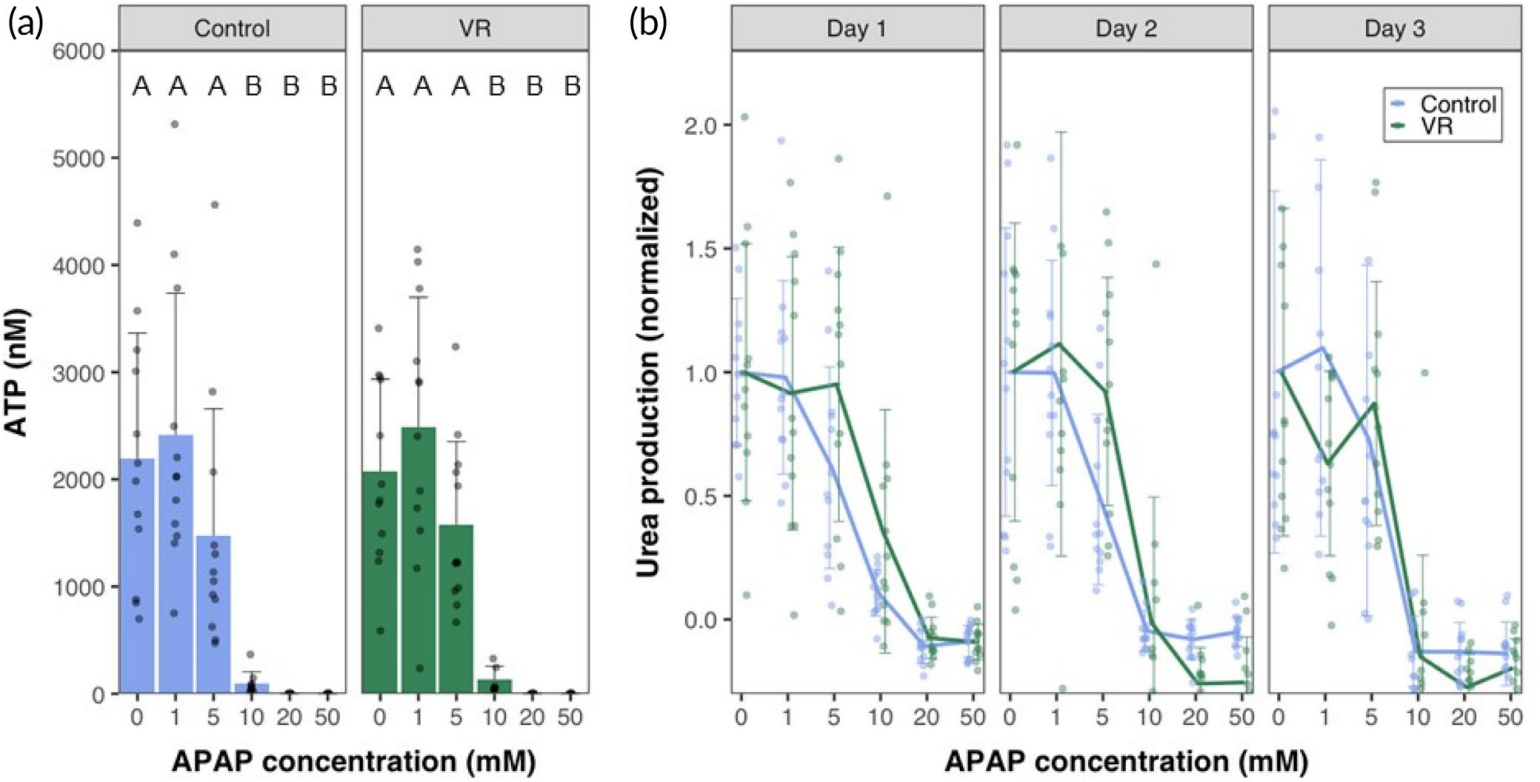
Drug response of precision‐cut liver slices. (a) ATP levels at the end of Day 3 in culture of control and vitrified and rewarmed (VR) slices exposed to varying concentrations of *N*‐acetyl‐para‐aminophenol (acetaminophen) (APAP). (b) Urea levels measured normalized to 0 mM concentration spanning a 3‐day culture period on exposure to different APAP concentrations. Data are mean ± standard deviation. Levels of significance are represented by compact letter display (Dunn's test).

Liver slices were exposed to increasing concentrations of APAP for 24 h. The APAP dosage was chosen based on previous studies in liver cell lines.^34^ Urea was measured in the same slices over 3 days in culture (Figure 6b). The slices were homogenized at the end of 72 h to measure ATP content (Figure 6a). VR and control slices performed very similarly, maintaining function and viability at APAP concentrations upto 5 mM, but 10 mM APAP resulted in severe injury and loss of function that increased over the culture period. At 20 and 50 mM APAP exposure, complete necrosis of slices occurred even on Day 1, with no urea detectable. The ATP content in the slices at the end of Day 3 also showed similar trends, with the slices exposed to 0–5 mM APAP maintaining ATP content while the slices exposed to 10–50 mM APAP showed undetectable ATP levels (below 25 nM). The ATP levels at Day 3 for the APAP study are lower than those shown in Figure 4a. The study in Figure 4a had dedicated slice groups for each measurement day, while the same set of slices was measured daily over 3 days in culture for the APAP study, requiring daily exposure to ammonium chloride. We posit that repeated exposure of the slices to high concentrations of ammonium chloride (10 mM) over the course of 3 days in culture led to some loss in viability.

## 4 DISCUSSION

Preventing drug failures during human clinical trials requires careful evaluation during the pre‐clinical stage. This capability can prevent patient harm, eliminate ineffective therapies, and ultimately increase successful outcomes while lowering costs. In the absence of in vivo testing, which is cost‐prohibitive and not fully accessible, cryopreserved liver tissue slices are a promising solution. PCLS banking from representative patient populations would open the possibility for repeatable drug screening and provide an accurate and patient‐representative model for studying diseases and their progressions. In this study, we illustrated in detail the first successful cryopreservation of rat liver slices through ice‐free VR to maintain viability, function, and enzymatic activity and show drug response post‐rewarming out to 3 days.

As mentioned, we focused on the culture of liver slices for 3 days, an acute testing window for a wide range of biomedical applications. Notably, no current standardized methodology for the culturing of liver slices exists. Many culturing methods involve using large Erlenmeyer flasks and culturing the slices in external oxygenated environments using carboxygen.^35^ Even with continuous improvement in culturing technology, the viability with most PCLS culture has only been reported for 24–48 h.^36^, ^37^ The most extended culture of PCLS was reported by Parish et al., who cultured PCLS for 6 days using a bioreactor,^8^ Rastovic et al., who cultured them for 6 days using an orbital shaker and Wu et al., who reported culturing PCLS for 15 days.^38^ However, the latter reported a decrease in specific gene expressions over the days in culture, which may indicate a transition to a non‐representative sample. Inspired by air‐interface culturing systems, we achieved a reproducible culture of liver slices for up to 3 days using the simple technique of suspending the PCLS on nylon meshes—this and optimizing the media height above the tissues allowed for better oxygen diffusion into the tissue. In comparison to literature, we report ATP^39^ and albumin values^8^ that are consistent with viable and functional PCLS over days in culture.

Having established a culture approach that can be reliably replicated, we turned to cryopreservation. Unsuccessful previous studies on liver slices employed slow rates of freezing and thawing (<2000°C/min cooling and rewarming) in cryovials using low concentrations of DMSO (18–22%v/v).^10^ These methods resulted in poor viability and function of slices even after short‐term culture. The poor viability could be attributed to disruption of the cell membranes due to ice formation and damage to the necessary extracellular microarchitecture disrupting normal function. Another major challenge with conventional methods for PCLS cryopreservation is a lack of reproducibility, given the limited control over where ice formation occurs. Vitrification circumvents these issues and allows for preserving PCLS for indefinite periods. Until now, limited attempts at VR liver slices have been reported,^40^ but the maintenance of viability and function of tissues over multiple days in culture has not been demonstrated.

Selecting the right CPA and protocol for loading and unloading was critical to succeed at VR, as injury can manifest in the form of osmotic injury and direct CPA toxicity.^41^ Osmotic injury occurs when the cells are exposed to sizeable transmembrane concentration gradients of the CPA, which causes acute shrinking of cells during loading and swelling of cells during unloading, leading to injury. One of the mechanisms of toxicity is the metabolization of the CPA components by the cells and their conversion into toxic byproducts. Osmotic injury can be minimized by controlled loading and unloading of the CPA, mitigating acute shrink‐swell behavior. We reduced toxicity by lowering the temperature of CPA exposure, which slows the metabolism of the components by the cells. Hence, we loaded the PCLS stepwise at 4°C to minimize injury and vitrified and rewarmed slices at high cooling and rewarming rates using a cryomesh. The VR slices demonstrated markers comparable to fresh slice controls for all metrics assessed in this study. This observed behavior was consistent for the VR slices over 3 days in culture. Slices stored for 60 days and rewarmed also showed consistent function over 3 days in culture, establishing our method as one that can enable “off‐the‐shelf” shipping in the future.

One of the critical differences that the PCLS provide as in vitro tools over cells is their ability to capture zonal activity during xenobiotic liver function. In this study, CYP1A1 activity was similar and sustained for control and VR PCLS for all days in culture. This has important implications for drug testing protocols and understanding the progression of liver diseases. The initiation and progression of liver diseases also occur in a zone‐wise manner, with many non‐alcoholic and alcoholic diseases of the liver beginning in Zone 3 and progressing to Zone 1.^42^ These zone‐dependent disease and signaling pathways cannot be studied using conventional cell‐based in vitro models.

The use of VR PCLS for drug hepatotoxicity was demonstrated with the common toxin, APAP, with demonstration of dose‐dependent toxicity similar to control PCLS. The response of the VR group to APAP was similar to controls, indicating no significant differences between the two groups on a macroscopic level over 3 days of culture. Most prior APAP toxicity testing outlined in the literature was performed using liver cell models. Increased toxicity levels have been reported at primarily high concentrations (>10 mM), but injury also occurs at lower concentrations (≤5 mM), where mitochondrial injury is thought to be the leading cause.^43^, ^44^, ^45^ In this PCLS study, there was no significant difference in ATP levels on APAP exposure from 0 to 5 mM in both groups. This indicates the opportunity to research the mechanisms of APAP toxicity using PCLS, which would more closely resemble in vivo whole liver conditions.

To summarize, this study used rat liver slices as a model system to establish successful VR using the cryomesh. Future studies will focus on obtaining human tissue to demonstrate success with both normal and diseased human liver slices. Overall, our study offers exciting opportunities to expand the use of PCLS in pharmacology and hepatology.

## AUTHOR CONTRIBUTIONS

SR contributed to conception, experimental design, data collection, data analysis, and manuscript writing. JSR contributed to obtaining AO/PI imaging data, B‐EN and MM contributed to the recovery of livers, BF and DKT contributed with assay data collection, DS, PM, and BA contributed to histological data interpretation, TLP contributed to experimental design, MLE contributed to experimental design and manuscript writing, JCB and EBF contributed to experimental design and manuscript writing and contributed as senior and corresponding authors.

## FUNDING INFORMATION

This work was supported by NIH grants DK117425 (Erik B. Finger, John C. Bischof) and DK132211 (Erik B. Finger, John C. Bischof), NSF grant EEC‐1941543 (Erik B. Finger, John C. Bischof, Timothy L. Pruett), and a gift from the Biostasis Research Institute funded in part through contributions from LifeGift, Nevada Donor Network, Lifesource, Donor Network West, and Lifebanc.

## CONFLICT OF INTEREST STATEMENT

John C. Bischof, Erik B. Finger, and Michael L. Etheridge disclose equity in a start‐up which is commercializing the cryomesh platform technology and related intellectual property (NorthStar Cryo, Inc.).

## Supporting information


**Data S1.** Supporting Information.

## Data Availability

All data supporting the findings of this paper are provided in the main text and in Supporting Information S1.
