# Supplementary material for: Vitrification and rapid rewarming of precision‐cut liver slices for pharmacological and biomedical research
Source: Bioeng Transl Med. 2025 Jul 17;11(1):e70045. doi: 10.1002/btm2.70045 (PMC12821208; doi:10.1002/btm2.70045)
Supplement: Supplementary file 1 — Data S1. Supporting Information. [file BTM2-11-e70045-s001.docx]

**Supplementary Information**

**Methods and Materials**

**Preparation of PCLS**

A 5 mm biopsy punch was used to obtain biopsies of the liver, followed by embedding of the biopsy onto the specimen tube using 3% (w/v) of low-melting agarose (IBI Scientific, IB70051) prepared in phosphate buffered saline (PBS). The specimen tube was then cooled using a chilling block to quickly set the agarose, followed by slicing using a Compresstome (Precisionary Instruments, MA, USA). The buffer tray of the Compresstome was filled with ice-cold PBS. The cut slices were collected in the tray, after which the agarose gel was removed, and the slices were placed in cold modified UW solution until further use (2-3 hours)

**Culture media composition**

The chemically defined culture media consisted of WilliamsE media (Gibco) supplemented with 10,000 ng/ml Insulin, 5.5 µg/ml Transferrin, 5 ng/ml Selenium (all Sigma), 2 mM L-glutamine (Sigma), 10 mM HEPES (Gibco), 50 µg/ml Gentamicin (Sigma), 2.5 µg/ml amphotericin-B (Sigma), and 0.1 µM Dexamethasone (Sigma). No serum was added to the culture media.

**Nylon meshes for culturing of PCLS**

Nylon meshes of 500 µm pore size and 0.3mm filament thickness were used for culturing. The meshes were initially cleaned with 70% Ethanol. After use, the meshes were washed in 10% Tergazyme detergent to remove any tissue remnants and then stored in 70% Ethanol under a bio safety cabinet.

**Thermometry of PCLS**

The cooling and rewarming temperatures were measured using a T-type fine gage bare wire thermocouple (COCO-002, OMEGA) and recorded using an oscilloscope (DS1M12, USB Instrument). Rates were then calculated from -100°C to -40°C and plotted as shown in Fig 2D.

**Assay assessments**

Assays were used for urea, albumin, and ATP as follows. For the assessment of urea, the slices were transferred to 24 well plates containing 0.25 ml of urea media for incubation times ranging from 1.5-3 hours. The urea media consisted of a KHB base solution with 10 mM ammonium chloride (Sigma Aldrich) and 2 mM L-ornithine (Sigma Aldrich) to assist in the initiation of the urea cycle. The slices were then either snap frozen, stored, and then homogenized for ATP assessment or put back into culture with fresh culture media in incubators at 37 °C with 5% CO_2_. For ATP, the slices were snap frozen and homogenized in 0.25ml of sonication buffer solution containing 70% (v/v) of Ethanol and 2mM EDTA (Ethylenediaminetetraacetic acid) at a pH of 10.9^35^. QuantiChrom Urea Assay Kit (BioAssay Systems) was used for the urea assay. Albumin was assessed from the culture media (1ml) using an ELISA assay (Rat albumin ELISA Kit, ICL). Finally, Roche Bioluminescence Assay Kit CLS II was used for ATP assay. All assays were performed according to the manufacturer’s instructions.

For AO/PI live/dead images presented in Fig.3, the PCLS were incubated with 8 ng/ml AO and 20 ng/ml PI (Millipore Sigma) for 5 min at room temperature. They were then imaged with an Olympus Fluoview 3000 inverted confocal microscope (Olympus) with 502/525-nm filters for AO and 493/636-nm filters for PI. The PCLS images were captured at 4,020 × 4,020-pixel resolution using a 20× magnification objective.

**Fixing, embedding and staining procedure**

PCLS were fixed in 10% Neutral Buffered Formalin. The slices were subsequently paraffin-embedded and 5µm sections were then taken close to the center of the slices and stained for Hematoxylin & Eosin (H&E) and TUNEL.

**Total protein analysis**

We also performed total protein analysis on the slices and found no significant difference between the protein amounts among the Control, CPA, VR, and FT groups. The Dead group shows lower protein, which can be explained by the loss of protein to the culture media during culturing due to extreme membrane damage and tissue degradation caused by freezing and thawing. Also note that the FT group could potentially have higher protein values than what is measured owing to similar damage caused by freezing and thawing. The protein content would also vary by slice since ice formation is uncontrolled during freezing and thawing.


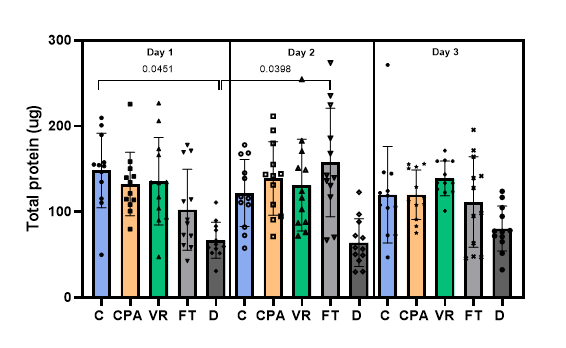


**Image analysis using ImageJ**

With ImageJ, we obtained a relative viability measure by counting the average number of dead cells (stained with red nuclei) to the total area by measuring the average area of each cell and allowing to estimate the number of cells in the given z-plane by excluding the central vein area and dividing the total area by the area of each cell. This was then followed by converting the red channel (PI signal) to 8-bit, followed by thresholding and counting the number of red nuclei using particle analysis, setting a threshold of 10 pixel^2^ area. The membrane integrity is presented as a percent using this image analysis method in Fig.3C.

**CYP1A1 (Resorufin production) live imaging and quantification**

For live imaging of CYP1A1 activity, the slices were incubated with culture media containing 25 µM β-naphthaflavone made in DMSO with 2.1 (v/v)% final DMSO concentration) for 24 hours to induce CYP1A1. They were then incubated with WilliamE media (no phenol red), 20 µM 7-Ethoxyresorufin, and 25 µM Dicumarol for 10 minutes and imaged using an excitation wavelength of 561nm laser in a Nikon A1RMP+ microscope. The CYP1A1 cleaves the 7-Ethoxyresorufin to fluorescent resorufin that can be imaged and quantified^22^. For quantification, the slices were placed in a microplate reader (Synergy HT, BioTek) and imaged for 30 minutes in kinetic mode using 535/595 nm filters at 37°C.

**Reagents and Equipment:**

| **Equipment/Reagent** | **Company** | **Location** |
| --- | --- | --- |
| Compresstome  Model VZ-310-0Z | Precisionary Instruments | Ashland, Massachusetts, USA |
| β-naphthaflavone | Cayman Chemical Company | Ann Arbor, Michigan, USA |
| 7-Ethoxyresorufin |  |  |
| Dicumarol |  |  |
| Acetaminophen |  |  |
| ETFE Cryomesh  Cat# 64700-24 | Electron Microscopy Sciences | Hatfield, Pennsylvania, USA |
| Nylon culturing mesh Cat #9318T44 | McMaster Carr | Elmhurst, Illinois, USA |
| Microplate reader  Synergy HT | BioTek | Winooski, Vermont, USA |
